# Effects of Maternal Nutrition and One-Carbon Metabolite Supplementation on Fetal Jejunal Morphology and Hexose Transporter Expression in Beef Cattle

**DOI:** 10.3390/vetsci12090884

**Published:** 2025-09-13

**Authors:** Mojtaba Daneshi, Pawel P. Borowicz, Virginia Montgomery, Yssi L. Entzie, Jessica G. Syring, Layla E. King, Kazi Sarjana Safain, Muhammad Anas, Lawrence P. Reynolds, Alison K. Ward, Carl R. Dahlen, Matthew S. Crouse, Joel S. Caton

**Affiliations:** 1 Department of Animal Sciences, Center for Nutrition and Pregnancy, North Dakota State University, Fargo, ND 58108, USA; 2 Department of Agriculture and Natural Resources, University of Minnesota Crookston, Crookston, MN 56716, USA; 3 Department of Veterinary Biomedical Sciences, University of Saskatchewan, Saskatoon, SK S7N 5B4, Canada; 4 United States Department of Agriculture, Agriculture Research Service, U.S. Meat Animal Research Center, Clay Center, NE 68933, USA

**Keywords:** developmental programming, early gestation, epigenetic modifications, folate, fetal intestine, glucose transporters, nutrient absorption, nutrition restriction, one-carbon metabolism

## Abstract

The objective of this study was to evaluate the effects of maternal nutrient restriction and one-carbon metabolite (OCM) supplementation on jejunal morphology and the abundance of hexose transporters (SLC2A1, SLC2A2, SLC2A3, SLC2A5, and SLC5A1) in bovine fetal jejunum. Twenty-nine crossbred Angus beef heifers were estrus-synchronized and artificially inseminated with female-sexed semen, then randomly assigned to one of four treatments in a 2 × 2 factorial design with two main factors: nutritional plane [control (CON) vs. restricted feed intake (RES)] and OCM supplementation [without OCM (−OCM) or with OCM (+OCM)]. From day 0 to 63 of gestation, CON heifers were fed to gain 0.45 kg/d, and RES heifers were fed to lose 0.23 kg/d. After d 63, all heifers received a common diet targeting a gain of 0.45 kg/d until d 161, when they were slaughtered for tissue collection and analysis. Hexose transporter abundance was evaluated via immunofluorescence and image analysis. Restricted maternal nutrition increased (*p* = 0.005) fetal villous height, whereas crypt depth was influenced (*p* = 0.02) by maternal nutrition × OCM supplementation, with RES + OCM showing deeper crypts. Muscle layers thicknesses also tended (*p* ≤ 0.08) to be greater in the RES + OCM. One-carbon metabolite supplementation decreased (*p* < 0.003) villus and total SLC2A1 abundance and tended (*p* = 0.09) to interact with nutrition in the crypts, with SLC2A1 levels being lowest in the CON + OCM. Abundance of SLC2A2 was influenced (*p* = 0.04) by an interaction, with RES − OCM showing reduced villus and total abundance. Villus and total SLC2A3 abundance increased (*p* = 0.08) in RES compared to CON groups but was reduced (*p* < 0.0001) in crypts with OCM supplementation. Villus SLC2A5 was affected (*p* = 0.05) by an interaction, being greatest in the RES + OCM, whereas crypt abundance was less (*p* = 0.03) in +OCM compared to −OCM. An interaction was observed for SLC5A1 (*p* ≤ 0.02), with the least villus and total abundance in CON – OCM, whereas crypt abundance was reduced in RES + OCM. These findings highlight the adaptive nature of the fetal small intestine to maternal dietary stress and indicate that OCM supplementation may help optimize fetal glucose metabolism and nutrient absorption.

## Simple Summary

This article is a revised and expanded version of a paper entitled “Impact of maternal nutrition and one-carbon metabolite supplementation during early pregnancy on glucose transporters in the bovine fetal intestine”, which was presented at the 2025 American Society of Animal Science (ASAS)-Midwest Section, Omaha, Nebraska, USA, 9–12 March 2025. The current manuscript provides a comprehensive analysis of additional transporters (SLC2A2, SLC2A5, SLC5A1) and morphology of small intestine, detailed methodology, extended results, and in-depth discussion, which were not included in the original abstract. Therefore, this submission does not constitute dual publication. Maternal nutrition during early pregnancy plays a vital role in the development of the fetus, shaping health and productivity outcomes later in life. Poor maternal nutrition can impair the structure and function of the fetal intestine, which is critical for nutrient absorption and energy metabolism. One-carbon metabolism, involving compounds like methionine, folate, choline, and vitamin B_12_, contributes to fetal development by supporting epigenetic changes that regulate gene expression. This study investigated how maternal nutrient restriction and one-carbon metabolite (OCM) supplementation affect the fetal jejunum, a key region of the small intestine responsible for nutrient uptake. We found that maternal nutrient restriction increased villus height, while OCM supplementation enhanced intestinal muscle thickness and modulated hexose transporter abundance, which may optimize nutrient absorption and supporting fetal adaptation to restricted nutrition. These findings highlight the importance of maternal nutrition and targeted supplementation strategies to improve livestock health and efficiency.

## 1. Introduction

The small intestine, particularly the jejunum, is a key site for nutrient absorption and metabolic activity in mature and growing ruminants. While the rumen is the primary site of starch digestion in ruminants, a variable portion of dietary starch—ranging from 4% to 60% depending on grain source, processing, and diet composition—can bypass ruminal fermentation and reach the small intestine, where it is digested and absorbed [1]. This process generates glucose and other monosaccharides—critical energy substrates—that are absorbed directly into the bloodstream [2,3,4]. The expression of specialized membrane proteins belonging to the solute carrier (SLC) family in the intestine is essential for the absorption of glucose, fructose, and other nutrients vital for energy metabolism and growth [5,6]. Among these, solute carrier family 5 member 1 (SLC5A1)—a sodium-dependent glucose cotransporter—and solute carrier family 2 member 2 (SLC2A2)—a facilitated glucose transporter—are the predominant glucose transporters in the small intestine, playing pivotal roles in monosaccharide absorption [7,8,9]. In contrast, SLC2A1 and SLC2A3, both high-affinity facilitated glucose transporters, are expressed at minimal levels in enterocytes [9,10,11] but are abundantly present in capillary endothelial cells, where they mediate glucose transport from the bloodstream into tissues [12,13,14]. Additionally, solute carrier family 2 member 5 (SLC2A5) is a fructose-specific facilitated transporter predominantly expressed in the small intestine and localized to both apical and basolateral membranes of enterocytes, facilitating fructose uptake from the intestinal lumen [9,15]. In addition to the critical role of hexose transporters, the morphology of the jejunum significantly contributes to its functionality. Villus height, a key determinant of the absorptive surface area, is positively associated with nutrient uptake and growth performance [16,17]. The thickness of the muscularis externa layer, composed of inner circular and outer longitudinal muscle layers, is crucial for maintaining motility and overall intestinal function [18,19]. By day 161 of gestation, which corresponds to the late second trimester in cattle, the fetal small intestine undergoes significant structural and functional maturation—including elongation of villi, deepening of crypts, increased vascularization, and upregulation of nutrient transporter expression—critical steps in preparing the intestine for extrauterine life [20]. However, the developmental trajectory of the fetal intestine differs significantly from that of postnatal animals.

Emerging evidence suggests that these morphological and functional factors in the small intestine are influenced while in utero by the maternal nutritional plane during pregnancy. Prenatal conditions may leave lasting imprints on the structure and function of the fetal small intestine as a consequence of developmental programming [20,21]. Developmental programming is the concept that various stressors, including compromised maternal nutrition during critical developmental windows, induce both short- and long-term changes in the offspring [22,23]. These changes span a wide range, including effects on growth [24], gene expression [25], immune system function [26], organ morphology [27], and metabolism [28]. For example, previous studies from our group demonstrated that restricted maternal nutrition during early pregnancy leads to increased capillary area density in the intestinal villi of bovine fetuses [29].

The most likely way that maternal nutrition influences fetal development is through epigenetic modifications [30,31]. One-carbon metabolites (OCM), such as folate, vitamin B_12_, methionine, and choline, are essential contributors to epigenetic regulation in the fetus, primarily through their roles in DNA and histone methylation [32,33]. By providing one-carbon units to the methionine and folate cycles, these metabolites facilitate the production of S-adenosylmethionine (SAM), a universal methyl donor. SAM is essential for DNA, RNA, and histone methylation, processes that regulate gene expression and chromatin structure. This intricate metabolic network adjusts to varying nutrient availability, ensuring cellular homeostasis and linking maternal nutrition to epigenetic status in the developing fetus. Enzymes such as DNA methyltransferases and histone methyltransferases use SAM to establish methylation marks that activate or silence genes in a tissue- and time-specific manner, shaping developmental outcomes [32,34,35].

Previous studies from our group have demonstrated that OCM supplementation can modulate the innate immune system [36] and influence weight and cell proliferation in the bovine fetal small intestine [29]. However, the impact of maternal nutrition and strategic OCM supplementation on the abundance of intestinal hexose transporters remains to be examined. Therefore, the aim of this study was to investigate how restricted maternal nutrition during early gestation, with or without OCM supplementation, affects the abundance of hexose transporters in the bovine fetal small intestine. We hypothesized that nutrient restriction would decrease hexose transporter abundance, while OCM supplementation would at least partially counteract these effects. The findings will enhance our understanding of the relationship between maternal nutrition and hexose transporter expression in the bovine fetus and evaluate the potential of methyl-donor supplementation to alleviate the negative impacts of poor maternal nutrition, ultimately supporting better developmental outcomes.

## 2. Materials and Methods

### 2.1. Animal Ethics

All experimental procedures were conducted in accordance with the guidelines established by the Institutional Animal Care and Use Committee at North Dakota State University (IACUC #20220059).

### 2.2. Experimental Design and Animal Management

A total of 72 Angus heifers were relocated from the Central Grasslands Research Extension Center in Streeter, ND, to the Animal Nutrition and Physiology Center at North Dakota State University in Fargo, ND. Before the study began, the heifers underwent a 14 d acclimation period to adjust to the feeding system. Following this adaptation, a 7 d Select Synch + CIDR protocol [37] was implemented for estrus synchronization. Artificial insemination was performed 18 to 22 h after estrus detection using female-sexed semen from a single sire (Connealy Maternal Made, ST Genetics, Navasota, TX, USA) to evaluate the effects of maternal nutrition and OCM supplementation on fetal development and to avoid sex-specific variation [38,39]; however, we acknowledge that it may limit the applicability of findings to male fetuses. All 72 heifers were assigned to treatments in a 2 × 2 factorial design at the start of the study. Of these, 29 heifers were later confirmed pregnant (~14 months old, 436 ± 42 kg initial body weight) with heifer fetuses and carried them to day 161 of gestation, at which point fetal tissues were collected for analysis. The two factors were nutritional plane [control (CON) vs. restricted (RES)] and OCM supplementation [without OCM (−OCM) or with OCM (+OCM)], resulting in four dietary treatment groups: CON − OCM (*n* = 7), CON + OCM (*n* = 8), RES − OCM (*n* = 7), and RES + OCM (*n* = 7). Individual feeding was conducted daily at 0800 h using an electronic head gate system (American Calan; Northwood, NH, USA). Pregnancy was confirmed on d 35 post-insemination via transrectal ultrasonography by detecting fetal heartbeats, and fetal sex was determined on d 63 using ultrasonography [40]. Only heifers carrying female fetuses were retained for subsequent study phases.

### 2.3. Dietary Treatments and Feeding Management

The CON diet was designed to meet 100% of the National Academy of Sciences, Engineering, and Medicine (NASEM) [41] nutritional requirements, targeting an average daily gain (ADG) of 0.45 kg/d. This regimen aimed to bring heifers to 80% of their mature body weight by calving. In contrast, heifers on the RES diet were fed to lose approximately 0.23 kg/d, simulating the natural production responses of heifers in a rangeland environment, who face dietary and environmental stress during early gestation [41]. Actual performance data from this study (unpublished) showed that heifers in the CON − OCM and CON + OCM groups achieved ADGs of 0.72 and 0.65 kg/d, respectively, while those in the RES − OCM and RES + OCM groups experienced average daily weight losses of 0.13 and 0.14 kg/d, respectively. The diets consisted of a total mixed ration of corn silage, alfalfa hay, corn grain, mixed alfalfa/grass hay, and a vitamin/mineral premix (Trouw Dairy VTM w/Optimins, Trouw Nutrition USA, Highland, IL, USA), with complete nutritional composition outlined in prior research [42]. Diets were tailored to meet the study’s objectives, providing 2.25 Mcal/kg ME, 9.75% CP, and 58.6% NDF. Individual feed rations were adjusted weekly based on body weight measurements to achieve the desired weight changes.

OCM-supplemented heifers (+OCM) received daily doses of 7.4 g of rumen-protected methionine (Smartamine, Adisseo, Beijing, China) and 44.4 g of rumen-protected choline (ReaShure, Balchem Inc., New Hampton, NY, USA), integrated into a corn carrier, in line with previous methodologies [43,44]. In addition, heifers received weekly intramuscular injections of vitamin B_12_ (cyanocobalamin; VetOne, MWI Animal Health, Boise, ID, USA) and folic acid (Spectrum Chemical Mfg. Corp., New Brunswick, NJ, USA), providing 20 mg of cyanocobalamin and 320 mg of folic acid per week, respectively, in accordance with established protocols [44,45]. Non-OCM-supplemented heifers (−OCM) received only the corn carrier without supplementation and were administered weekly intramuscular saline injections. These treatment protocols continued until d 63 of gestation, after which all heifers were managed on the CON − OCM treatment, targeting a daily gain of 0.45 kg for the remainder of the study.

### 2.4. Sample Collection and Tissue Preparation

On d 161 ± 1 of gestation, heifers were slaughtered for sample collection. Fetal jejunum samples were obtained by first locating the third branch of the mesenteric vein where it extends from the portal vein, then following its arcade to where it enters the jejunal tissue. Jejunal samples were collected from the area just caudal to this insertion point [46]. The samples were initially fixed in 4% neutral buffered formalin (NBF) for 24 h, followed by transfer to 70% ethanol. For histological and subsequent image analyses, the tissues were processed by cutting cross sections 2–3 mm thick, progressively dehydrating them through increasing alcohol concentrations, clearing in xylene, and infiltrating with paraffin wax (Leica Biosystems Inc., Buffalo Grove, IL, USA), and embedding using Tissue-Tek TEC embed (Sakura Finetek, Torrance, CA, USA).

### 2.5. Immunofluorescence Staining

Tissue sections, prepared at a thickness of 5 μm, underwent immunostaining. Slides were deparaffinized using xylene and rehydrated through a graded series of alcohols. Antigen retrieval was performed by heating sections in 0.01 M sodium citrate buffer (pH 6) in a pressure cooker for 20 min, followed by cooling at room temperature (approximately 23 °C) for 10 min and a 3 min wash with Tris-buffered Saline, 0.2% Tween 20 (TBST). To minimize nonspecific antibody binding, sections were blocked with 5% normal goat serum for 1 h at room temperature. The details of the primary antibodies used are provided in Table 1, with their optimal concentrations established through preliminary testing on various tissues. Incubation with primary antibodies was performed in a humidified chamber, with SLC2A1, SLC2A2, and SLC5A1 incubated for 1 h at room temperature, while SLC2A3 and SLC2A5 were incubated overnight at 4 °C. Afterward, sections were washed with TBST for 3 min and incubated with a fluorochrome 633 (Biotium, Fremont, CA, USA) goat anti-rabbit secondary antibody for 1 h at room temperature, except for SLC5A1 and SLC2A5, for which fluorochrome 555 and 568 (Biotium), respectively, were used. The staining process concluded with nuclear counterstaining using 4′,6-diamidino-2-phenylindole (DAPI, 1%, Biotium) for 5 min.

### 2.6. Image Analysis

High-resolution, large-area mosaic images of complete jejunal cross-sections were captured using a Leica Mica microscope (Leica Microsystems, Wetzlar, Germany) in Thunder mode with a PL APO 20 × 0.75 NA objective. Consistent exposure time and energy settings were applied across all slides to ensure comparability. For each slide, three distinct areas were imaged from the villi and the crypts.

**Figure 1:**
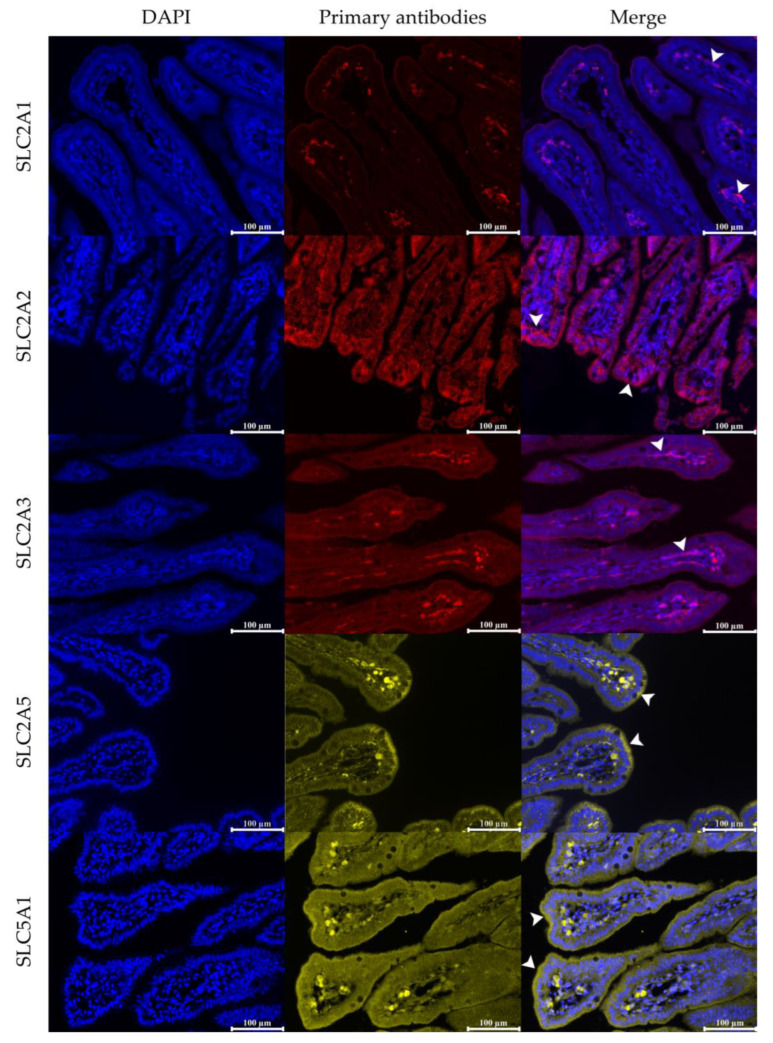
Immunofluorescence images of hexose transporter proteins (SLC2A1, SLC2A2, SLC2A3, SLC2A5, and SLC5A1) in the fetal jejunum. Sections were stained using specific primary antibodies, followed by Biotium secondary antibodies: Biotium 633 for SLC2A1, SLC2A2, SLC2A3, Biotium 568 for SLC2A5, and Biotium 555 SLC5A1. Nuclei were counterstained with DAPI. Representative images show the localization (indicated by white arrowheads) and relative abundance of transporters in villi. All images are at 200× magnification. The white scale bar on each image represents 100 μm.

The acquired mosaic images were subjected to detailed analysis using Image Pro Premier v. 9.1 (Media Cybernetics, Rockville, MD, USA). Regions of interest within the cross-sections were precisely delineated and analyzed for fluorescence intensity, with results quantified as relative fluorescence units, serving as an indicator of transporters abundance. The transporter positivity ratio (Figure 1) was calculated as the ratio of positively stained areas to the total area within each region of interest [29]. Additionally, villus height, crypt depth, as well as the thickness of inner circular and outer longitudinal muscles layers were measured by computerized image analysis in 3 random images per slide [46,47]. These images covered at least 1:4 distinct slices on the slide. All well-oriented and complete structures, including all villi directly connected to the crypts, were measured.

### 2.7. Statistical Analysis

Data analysis was performed using the PROC MIXED procedure of SAS v. 9.4 (SAS Institute Inc., Cary, NC, USA). Before analysis, box plots were generated with PROC SGPLOT using the VBOX statement to visually assess the data. Post-analysis, the normality of residuals was evaluated using the Shapiro–Wilk test in PROC UNIVARIATE, followed by QQ plots. Fixed effects in the model included nutritional level, OCM treatment, and their interaction. Individual heifers were treated as experimental units, and images were modeled as a repeated measure within each heifer, with an unstructured covariance matrix specified using the TYPE = UN statement to account for within-subject correlations. If no significant interactions were detected, the main effects of maternal nutrition and OCM treatment were reported. Pairwise comparisons of means were conducted using the PDIFF function with Tukey–Kramer adjustment, and results were presented as least squares means (LSMEANS) with their standard errors (SEM). The largest SEM is noted. Statistical significance was determined at *p* ≤ 0.05, with trends reported for 0.05 < *p* ≤ 0.1.

## 3. Results

### 3.1. Morphology of the Small Intestine

Villus height was not influenced (*p* = 0.97) by the interaction between maternal nutrition and OCM supplementation. However, maternal nutrition independently affected villous height (*p* = 0.005), with the RES treatment exhibiting a 7.95% increase compared with the CON treatment (Table 2). Crypt depth was influenced (*p* = 0.02) by the interaction between maternal nutrition and OCM supplementation, where the RES + OCM treatment exhibited deeper crypts compared to the CON + OCM (*p* = 0.02) and RES − OCM treatments (*p* = 0.04; Table 2). The villus height-to-crypt depth ratio was unaffected by maternal nutrition, OCM supplementation, or their interaction (*p* ≥ 0.15; Table 2).

Inner circular muscle thickness exhibited a trend (*p* = 0.08) for an interaction between maternal nutrition and OCM supplementation, where the RES + OCM treatment showed greater thickness compared to the CON − OCM (*p* = 0.07), CON + OCM (*p* = 0.01), and RES − OCM treatments (*p* = 0.06; Table 2). Similarly, outer longitudinal muscle thickness tended (*p* = 0.06) to be influenced by the interaction, with the RES + OCM treatment having greater thickness than the CON − OCM (*p* = 0.08), CON + OCM (*p* = 0.01), and RES − OCM treatments (*p* = 0.06). Total muscle layer thickness followed a similar trend (*p* = 0.06), where the RES + OCM treatment had increased thickness compared to the CON − OCM (*p* = 0.02), CON + OCM (*p* < 0.01), and RES − OCM treatments (*p* = 0.04; Table 2).

### 3.2. Relative Protein Abundance of SLC2A1

Villus SLC2A1 abundance was not influenced (*p* = 0.58) by the interaction between maternal nutrition and OCM supplementation. However, OCM supplementation independently reduced villus SLC2A1 abundance (*p* = 0.003), with the −OCM treatment exhibiting greater levels than the +OCM treatment (Table 3). Crypt SLC2A1 abundance exhibited a trend (*p* = 0.09) for interaction between maternal nutrition and OCM supplementation, where the CON + OCM treatment had lower abundance compared to the CON − OCM (*p* = 0.0009), RES − OCM (*p* = 0.0003), and RES + OCM treatments (*p* = 0.007; Table 3). For total SLC2A1 abundance, OCM supplementation reduced overall levels (*p* = 0.001), while no interaction (*p* = 0.48) was observed between maternal nutrition and OCM supplementation (Table 3).

### 3.3. Relative Protein Abundance of SLC2A2

Villus SLC2A2 abundance was affected (*p* = 0.04) by the interaction between maternal nutrition and OCM supplementation. Specifically, the RES − OCM treatment exhibited lower SLC2A2 abundance compared with the CON − OCM treatment (*p* = 0.007; Table 4). Total SLC2A2 abundance was influenced (*p* = 0.04) by the interaction between maternal nutrition and OCM supplementation, where the RES − OCM treatment exhibited lower levels compared to the CON − OCM (*p* = 0.005; Table 4).

### 3.4. Relative Protein Abundance of SLC2A3

No interaction (*p* = 0.23) was observed between maternal nutrition and OCM supplementation for the protein abundance of SLC2A3 in villi. However, a trend (*p* = 0.08) was noted, with the RES treatment exhibiting higher levels than the CON treatment (Table 5). In the crypts, no interaction (*p* = 0.84) was detected between maternal nutrition and OCM supplementation. However, crypt SLC2A3 abundance was influenced (*p* < 0.0001) by OCM supplementation, where the −OCM treatment showed greater levels than the +OCM treatment (Table 5). Total SLC2A3 abundance was not affected (*p* = 0.23) by the interaction between maternal nutrition and OCM supplementation (Table 5). However, a trend was observed for the nutritional level (*p* = 0.08), with the RES treatments showing greater SLC2A3 levels compared to the CON treatments (Table 5).

### 3.5. Relative Protein Abundance of SLC2A5

An interaction (*p* = 0.05) between maternal nutrition and OCM supplementation was observed for villus SLC2A5 protein abundance. Notably, the RES + OCM treatment showed a greater SLC2A5 abundance compared to the CON − OCM (*p* = 0.02), CON + OCM (*p* = 0.02), and RES − OCM treatments (*p* = 0.008; Table 6). In the crypts, no interaction (*p* = 0.17) between maternal nutrition and OCM supplementation was detected. However, crypt SLC2A5 abundance was affected (*p* = 0.03) by OCM supplementation, with the +OCM treatment showing a 24% reduction compared to the −OCM treatment (Table 6). Total SLC2A5 abundance did not differ between treatments (*p* > 0.28; Table 6).

### 3.6. Relative Protein Abundance of SLC5A1

An interaction (*p* = 0.01) was observed between maternal nutrition and OCM supplementation for the protein abundance of villus SLC5A1. Specifically, the CON − OCM treatment exhibited lower SLC5A1 protein abundance compared to the CON + OCM (*p* = 0.002), RES − OCM (*p* < 0.0001), and RES + OCM (*p* < 0.0001; Table 7) treatments. Crypt SLC5A1 abundance was influenced (*p* = 0.02) by the interaction between maternal nutrition and OCM supplementation, where the RES + OCM treatment exhibited lower levels compared to the CON − OCM (*p* = 0.005), CON + OCM (*p* = 0.0005), and RES − OCM (*p* = 0.01; Table 7) treatments. Total SLC5A1 abundance was also influenced (*p* = 0.003) by the interaction, where the CON − OCM treatment had the lowest levels compared to the CON + OCM (*p* = 0.002), RES − OCM (*p* < 0.0001), and RES + OCM treatments (*p* = 0.004; Table 7).

## 4. Discussion

This study investigated the effects of maternal nutrient restriction during early gestation, with or without OCM supplementation, on the morphology and abundance of hexose transporters in the bovine fetal small intestine. The findings highlight several critical insights into the interplay between maternal nutrition and supplementation, advancing our understanding of how these factors influence fetal intestinal growth and adaptation.

### 4.1. Morphological Adaptations of the Fetal Small Intestine

In our study, we observed that villus height was greater in the RES compared with the CON treatment. This finding aligns with observations from previous studies [27,48], suggesting an adaptive response to enhance the absorptive surface area of the intestine This adaptation should enable the fetus to maximize nutrient uptake under conditions of limited maternal nutrient supply. Supporting this, our previous study within the same experimental framework [29] found greater capillary area density and spatial cell density in the jejunal villi of the RES treatment, further indicating a compensatory mechanism for optimizing nutrient absorption. Such changes may reflect fetal programming, where the fetus adjusts its development in response to maternal signals indicating anticipated environmental conditions [20]. In contrast, studies in ovine [21] and bovine [46] models have reported no significant differences in villus height between restricted and control treatments. These discrepancies could be attributed to differences in the timing and severity of nutrient restrictions [20]. In the present study, nutritional treatments were initiated at breeding, targeting a critical window of early fetal development. Furthermore, the moderate severity of restriction in the present study may have allowed sufficient substrate availability to support compensatory growth mechanisms, unlike in studies with more severe restrictions.

Crypt depth, as well as the thickness of the inner circular and outer longitudinal muscle layers, were influenced by the interaction between maternal nutrition and OCM supplementation. Specifically, the RES + OCM treatment exhibited greater values for these parameters compared to other treatments. Crypt depth is closely associated with crypt and villus cell proliferation, which is crucial for continuous epithelial renewal. A previous study demonstrated that a deficiency in methyl donors (including vitamin B_12_, folate, and choline) during gestation and lactation leads to increased apoptosis in the intestinal crypts and reduced epithelial cell differentiation in the villi. This effect was attributed to elevated plasma homocysteine levels, which are known to induce apoptosis by causing DNA damage and impairing DNA repair mechanisms [49]. Interestingly, our group previously found that nutrient-restricted pregnant heifers had greater serum homocysteine levels [50], and observed an inverse correlation between maternal serum homocysteine and fetal amniotic fluid folate concentration [51]. Therefore, the increased crypt depth observed with OCM supplementation may suggest a mitigating effect against villus atrophy caused by undernutrition [52,53].

To the best of our knowledge, this is the first study to evaluate the thickness of the muscularis externa of the bovine fetal small intestine, composed of the inner circular and outer longitudinal muscle layers. This assessment is critical for understanding the development of the small intestine, as these muscle layers play a key role in intestinal motility and efficient nutrient transport and absorption. The observed increase in muscle layer thickness in the RES + OCM suggests that OCM supplementation may exert a protective effect during periods of nutrient restriction. Thicker muscular layers suggest enhanced intestinal motility, which is crucial for effective digestion and nutrient absorption [18,19]. A recent study reported that dietary folic acid supplementation during pregnancy significantly increased muscle layer thickness in newborn lambs [54]. Similarly, we previously showed that OCM supplementation during early gestation promotes allometric growth of fetal cardiac and skeletal muscle tissue [55]. Moreover, prior findings from our lab demonstrated that nutrient restriction in pregnant heifers reduces methionine concentrations in allantoic fluid [50], while OCM supplementation increases the abundance of metabolites involved in pathways associated with methionine [56]. Methionine plays a vital role in protein synthesis and DNA methylation–processes that are essential for proper fetal growth [57], and as such, OCM supplementation may counteract the adverse effects of nutrient restriction by restoring methionine availability and mitigating the negative impacts on intestinal development and function. However, increased muscle thickness may raise the intestinal tissue’s metabolic demands due to higher energy and oxygen needs for maintaining smooth muscle function. Prezotto et al. [58] reported increased fetal small intestinal oxygen consumption in fetuses from nutrient-restricted and realimented dams, suggesting possible trade-offs. Future studies should quantify energy use across intestinal compartments to better understand these adaptations.

### 4.2. Hexose Transporters of the Small Intestine

Alterations in hexose transporters expression in the fetal small intestine may have lasting consequences beyond gestation, potentially impairing intestinal absorptive function and postnatal growth. This notion is supported by findings in both rodent and ruminant models [59,60], which demonstrate that early-life nutritional insults can program long-term changes in intestinal structure, gene expression, and metabolic capacity. These insights underscore the physiological relevance of assessing transporter expression during fetal development and its implications for neonatal health.

#### 4.2.1. The Abundance of SLC2A1 and SLC2A3 Proteins

SLC2A1 and SLC2A3 are high-affinity glucose transporters predominantly expressed in capillary endothelial cells, facilitating glucose uptake into tissues [12,13,14]. SLC2A1, in particular, plays a crucial role in maintaining basal glucose transport across blood-tissue barriers and supporting energy demands, while SLC2A3 is associated with cells requiring rapid bursts of energy [61,62,63].

In the present study, the abundance of SLC2A1 was generally lower in the +OCM treatment, while SLC2A3 exhibited upregulation in fetuses from RES dams. The lower abundance of SLC2A1 in OCM-supplemented groups may indicate a shift toward enhanced metabolic efficiency, reducing reliance on high-affinity glucose transporters, as it has been shown that elevated plasma glucose levels tend to suppress SLC2A1 expression in the jejunum [64]. One-Carbon metabolite supplementation increases glucose and fructose concentrations in fetal fluids of RES heifers [65] and supports energy and carbohydrate metabolism pathways during nutrient-restricted conditions by increasing metabolites in the methionine, cysteine, SAM, and taurine metabolism pathways and enriching the disaccharides and oligosaccharides metabolism pathway [56]. These improvements likely reduce the need for high-affinity glucose transporters, as fetal nutrient utilization is more effectively supported in the OCM-supplemented environment, suggesting a programming effect on fetal metabolism. Additionally, chronic hypoxia, which often accompanies maternal nutrient deprivation due to reduced placental blood flow and oxygen delivery [66], is another factor known to upregulate SLC2A1 and SLC2A3 [14,67,68]. Our previous findings showed that OCM supplementation enhances placental vascularity, potentially improving uteroplacental blood flow [69]. This improvement may mitigate hypoxia-driven upregulation of SLC2A1. It is also worth mentioning that a recent study has linked hypermethylation of the SLC2A1 gene to decreased expression of SLC2A1 protein in brain tissues [70]. While we determined the optimal dosage of OCM supplementation in vivo [44], the potential effect of hypermethylation on SLC2A1 expression cannot be overlooked and warrants further investigation.

Conversely, the increased abundance of SLC2A3 observed in the RES treatment likely reflects a compensatory mechanism to ensure adequate glucose uptake under nutrient-limited conditions. This aligns with their high glucose affinity and critical role in maintaining energy homeostasis during maternal nutrient restriction [12]. Hypoglycemia is known to upregulate SLC2A1 [71] and SLC2A3 [72], optimizing glucose uptake when blood glucose levels are low, further supporting the observed response in the RES treatment. Interestingly, our laboratory previously demonstrated that restricted maternal nutrition reduces glucose levels in both amniotic and allantoic fluids [50], providing further context to the observed compensatory upregulation of SLC2A3 in fetuses from restricted dams.

#### 4.2.2. The Abundance of SLC2A2, SLC2A5, and SLC5A1 Proteins

Solute carrier family 2 member 2 (SLC2A2), SLC2A5 and SLC5A1 are the primary hexose transporters present in the small intestine. In this study, the abundance of these transporters was influenced by maternal nutritional levels and OCM supplementation, underscoring their adaptability to fetal nutritional programming.

Solute carrier family 2 member 2 is a low-affinity, high-capacity glucose transporter primarily localized to the basolateral membrane of enterocytes, where it facilitates glucose efflux into the bloodstream. In this study, the abundance of SLC2A2 was greater in fetuses from the RES + OCM treatment compared to RES − OCM treatment. Previous findings have shown that SLC2A2 expression is sensitive to glucose availability and dietary composition. Studies have demonstrated that SLC2A2 expression is regulated by luminal substrates and diurnal rhythms, with jejunal expression increasing during periods of maximal feeding [73,74]. Evidence indicates that a glucose-dependent increase in SLC2A2 abundance occurs at the brush border membrane (BBM) under high luminal glucose conditions, whereas its abundance decreases when luminal glucose levels drop [75,76]. Interestingly, OCM supplementation appeared to counteract these changes in this study, maintaining SLC2A2 levels even under restricted nutrition. This effect may stem from the role of OCM in providing methyl donors for DNA methylation, which is a key mechanism regulating SLC2A2 expression [77,78,79]. By supporting DNA methylation, OCM supplementation could modify the abundance of SLC2A2, potentially programming the fetus to adapt more effectively to future nutritional restrictions.

Solute carrier family 5 member 1 (SLC5A1), a sodium-dependent glucose cotransporter, is a high-affinity transporter that facilitates the active absorption of monosaccharides such as glucose and galactose, but not fructose [75]. The interaction between maternal nutrition and OCM supplementation influenced SLC5A1 expression. The abundance of SLC5A1 was greater in the CON + OCM treatment compared to the CON – OCM treatment, suggesting that under sufficient nutrient availability, OCM enhances SLC5A1 expression. This aligns with studies showing folic acid supplementation enhances intestinal SLC5A1 in offspring from well-nourished ewe [54]. Conversely, in the folate-deficient group, OCM supplementation did not significantly alter SLC5A1 abundance [80], indicating that the physiological response to nutrient restriction may override modulatory effects of OCM. This could be attributed to the already elevated baseline expression of SLC5A1 in the RES − OCM, driven by a compensatory mechanism to maximize glucose absorption.

Solute carrier family 2 member 5 (SLC2A5), a fructose-specific transporter localized to the brush border and basolateral membranes of small intestinal enterocytes, and facilitates passive fructose uptake from the intestinal lumen [75,81]. In ungulates, fructose is a major hexose sugar in fetal fluids and blood and serves as an important energy substrate during development. It has been shown to support fetal energy demands and stimulate trophoblast cell proliferation, at least in part through activation of the mammalian target of rapamycin (mTOR) signaling pathway, which regulates cell growth, metabolism, and protein synthesis [82]. In a previous study by our group, fructose concentrations in amniotic fluid were significantly decreased in nutrient-restricted heifers compared with controls [50], highlighting its sensitivity to maternal nutrition. In the present study, fetal jejunal SLC2A5 abundance was greatest in the RES + OCM group, suggesting that OCM supplementation may restore or enhance the fetus’s ability to absorb fructose under restricted conditions. Given the critical role of fructose in supporting fetal metabolism and activating nutrient-sensing pathways, this increase in SLC2A5 expression may have meaningful implications for fetal growth and development. Moreover, SLC2A5 expression is regulated by epigenetic mechanisms, including histone modifications [83,84] and DNA methylation [85], which may mediate the observed responses to maternal diet and supplementation.

Interestingly, crypt SLC2A5 abundance was lower in the +OCM treatment, irrespective of maternal nutritional status. This aligns with a broader pattern observed for other transporters in this study, where OCM supplementation, either independently or in combination with maternal nutrition, led to reduced transporter abundance in crypts (SLC2A1, SLC2A3, SLC2A5, and SLC5A1) but increased abundance in villi (SLC2A2, SLC2A5, and SLC5A1). Such a redistribution of hexose transporters suggests that OCM supplementation may program the fetus, likely via epigenetic pathways, to optimize nutrient absorption at the villus level. This adaptive mechanism could enhance absorption efficiency under diverse physiological and dietary conditions [75].

Our results highlight the adaptive nature of hexose transporters in response to maternal nutritional and supplementation strategies. As demonstrated by previous research, the expression of hexose transporters varies with developmental stage throughout gestation [86,87,88,89] and is influenced by dietary factors postnatally [79,90]. This highlights the importance of evaluating the expression of hexose transporters during late gestation, particularly before delivery, to better understand their role in fetal glucose metabolism and readiness for postnatal life. Furthermore, evaluating hexose transporters under different dietary conditions postnatally is recommended to gain insights into their adaptive responses and optimize nutritional strategies for early development. Although the present study did not directly assess functional activity, future studies should incorporate nutrient absorption assays to complement protein abundance data and provide a clearer picture of physiological relevance. Future studies should also incorporate quantitative assays such as Western blotting or ELISA to more precisely quantify protein expression levels and strengthen the interpretation of transporter abundance. Additionally, while we did not directly examine epigenetic regulation, future investigations should explore how maternal nutrition and OCM supplementation may influence epigenetic mechanisms such as DNA methylation and histone modifications affecting hexose transporter genes. Postnatal follow-up studies are also warranted to determine whether observed fetal changes persist after birth and contribute to improved growth and health outcomes. Finally, including male fetuses in future experimental designs will help assess sex-specific responses and enhance the generalizability of findings.

## 5. Conclusions

This study demonstrates that maternal nutritional restriction and OCM supplementation during early gestation have significant effects on the abundance and localization of hexose transporters in the bovine fetal jejunum at d 161 of gestation. The findings highlight a dynamic interplay between maternal nutrition and OCM supplementation in modulating hexose transporter proteins, suggesting potential epigenetic regulation. OCM supplementation was shown to counteract some adverse effects of maternal nutrient restriction, enhancing nutrient transporter abundance in villi while reducing it in crypts. This redistribution of transporter expression likely represents an adaptive programming mechanism to optimize nutrient absorption from the intestinal lumen.

## Figures and Tables

**Table 1:** Specifications of antibodies employed for Immunofluorescence in the study.

Antigen ^1^	Type	Dilution	Code	Supplier
SLC2A1	Rabbit, Monoclonal	1:1000	ab115730	Abcam, Boston, MA, USA
SLC2A2	Rabbit, Polyclonal	1:150	ab54460	Abcam, Boston, MA, USA
SLC2A3	Rabbit, Polyclonal	1:500	ab15311	Abcam, Boston, MA. USA
SLC2A5	Mouse, Monoclonal	1:400	Sc271055	Santa Cruz, CA, USA
SLC5A1	Rabbit, Polyclonal	1:100	PA5-28240	Invitrogen, Mt Prospect, IL, USA

^1^ SLC2A1–SLC2A5 = Solute carrier family 2 members 1 to 5; SLC5A1 = Solute carrier family 5 member 1.

**Table 2:** The effect of maternal nutritional levels [Nut; control (CON) or restricted (RES)] ^1^ and supplementation with one-carbon metabolites [OCM; with OCM (+OCM) or without (−OCM)] ^2^ from d 0 to 63 of gestation on the jejunal morphology of bovine fetuses at 161 days of gestation.

			Supplementation				*p*-Values			
Item	Nut		−OCM	+OCM	SEM ^3^	Nut ^4^	SEM ^5^	Nut	OCM	Nut × OCM
Villus height, μm	CON		354.69	365.39	10.10	360.04	7.08	0.005	0.27	0.97
	RES		383.00	394.02		388.68				
		OCM ^6^	368.84	379.88						
Crypt depth, μm	CON		100.2 ^ab^	94.61 ^a^	3.60	97.40	2.46	0.36	0.51	0.02
	RES		95.49 ^a^	105.69 ^b^		100.59				
		OCM	97.85	100.15						
Villus height/Crypt depth	CON		3.62	3.91	0.21	3.76	0.14	0.28	0.95	0.15
	RES		4.14	3.83		3.98				
		OCM	3.88	3.87						
Inner circular muscle thickness, μm	CON		54.40 ^A^	52.78 ^aAB^	2.05	53.59	1.40	0.09	0.35	0.08
	RES		54.28 ^A^	59.59 ^bB^		56.94				
		OCM	54.34	56.18						
Outer longitudinal muscle thickness, μm	CON		31.57 ^A^	30.60 ^aAB^	1.16	31.09	0.80	0.12	0.31	0.06
	RES		31.25 ^A^	34.48 ^bB^		32.85				
		OCM	31.40	32.54						
Total muscle layer thickness, μm ^7^	CON		85.33 ^a^	83.39 ^a^	2.75	84.36	1.90	0.03	0.26	0.06
	RES		86.07 ^a^	94.07 ^b^		90.07				
		OCM	85.70	88.73						

The data are presented as the least squares means and standard errors of the means (SEM). ^1^ CON (0.45 kg·heifer^−1^·d^−1^); RES = (−0.23 kg·heifer^−1^·d^−1^). ^2^ +OCM = (with one-carbon metabolites supplementation); –OCM = (without one-carbon metabolites supplementation). ^3^ Average SEM for nutritional levels × OCM supplementation interaction [CON − OCM (*n* = 7); CON + OCM (*n* = 8); RES − OCM (*n* = 7); RES + OCM (*n* = 7)]. ^4^ Main effect of nutritional levels. ^5^ Average SEM for Nut and OCM. ^6^ Main effect of OCM supplementation. Lowercase letters indicate significant differences (*p* ≤ 0.05), and uppercase letters denote trends (0.05 < *p* ≤ 0.10). ^7^ The total muscle layer thickness was calculated by adding the measured thicknesses of both the inner circular and outer longitudinal muscle layers.

**Table 3:** The effect of maternal nutritional levels [Nut; control (CON) or restricted (RES)] ^1^ and supplementation with one-carbon metabolites [OCM; with OCM (+OCM) or without (−OCM)] ^2^ from d 0 to 63 of gestation on the protein abundance of solute carrier family 2 member 1 (SLC2A1) in the fetal jejunum at 161 d of gestation.

			Supplementation				*p*-Values			
Area	Nut		−OCM	+OCM	SEM ^3^	Nut ^4^	SEM ^5^	Nut	OCM	Nut × OCM
Villus	CON		15.34	10.44	1.40	12.89	0.97	0.83	0.003	0.58
	RES		14.88	11.49		13.18				
		OCM ^6^	15.11	10.96						
Crypt	CON		1.96 ^a^	1.28 ^b^	0.14	1.62	0.09	0.03	0.002	0.09
	RES		2.03 ^a^	1.83 ^a^		1.93				
		OCM	1.99	1.55						
Total	CON		17.30	11.72	1.44	14.51	1.02	0.67	0.001	0.48
	RES		16.91	13.32		15.11				
		OCM	17.11	12.52						

Data are presented as relative fluorescence units. ^1^ CON (0.45 kg·heifer^−1^·d^−1^); RES = (−0.23 kg·heifer^−1^·d^−1^). ^2^ +OCM = (with one-carbon metabolites supplementation); −OCM = (without one-carbon metabolites supplementation). ^3^ Average SEM for nutritional levels × OCM supplementation interaction [CON − OCM (*n* = 7); CON + OCM (*n* = 8); RES − OCM (*n* = 7); RES + OCM (*n* = 7)]. ^4^ Main effect of nutritional levels. ^5^ Average SEM for Nut and OCM. ^6^ Main effect of OCM supplementation. Lowercase letters indicate significant differences (*p* ≤ 0.05).

**Table 4:** The effect of maternal nutritional levels [Nut; control (CON) or restricted (RES)] ^1^ and supplementation with one-carbon metabolites [OCM; with OCM (+OCM) or without (−OCM)] ^2^ from d 0 to 63 of gestation on the protein abundance of solute carrier family 2 member 2 (SLC2A2) in the fetal jejunum at 161 days of gestation.

			Supplementation				*p*-Values			
Area	Nut		−OCM	+OCM	SEM ^3^	Nut ^4^	SEM ^5^	Nut	OCM	Nut × OCM
Villus	CON		11.25 ^a^	10.29 ^ab^	0.62	10.77	0.44	0.06	0.66	0.04
	RES		8.88 ^b^	10.36 ^ab^		9.62				
		OCM ^6^	10.06	10.33						
Crypt	CON		0.19	0.17	0.02	0.18	0.01	0.54	0.48	0.68
	RES		0.17	0.16		0.17				
		OCM	0.18	0.16						
Total	CON		11.64 ^a^	10.51 ^ab^	0.63	11.07	0.45	0.04	0.76	0.04
	RES		9.03 ^b^	10.53 ^ab^		9.78				
		OCM	10.33	10.52						

Data are presented as relative fluorescence units. ^1^ CON (0.45 kg·heifer^−1^·d^−1^); RES = (−0.23 kg·heifer^−1^·d^−1^). ^2^ +OCM = (with one-carbon metabolites supplementation); –OCM = (without one-carbon metabolites supplementation). ^3^ Average SEM for nutritional levels × OCM supplementation interaction [CON − OCM (*n* = 7); CON + OCM (*n* = 8); RES − OCM (*n* = 7); RES + OCM (*n* = 7)]. ^4^ Main effect of nutritional levels. ^5^ Average SEM for Nut and OCM. ^6^ Main effect of OCM supplementation. Lowercase letters indicate significant differences (*p* ≤ 0.05).

**Table 5:** The effect of maternal nutritional levels [Nut; control (CON) or restricted (RES)] ^1^ and supplementation with one-carbon metabolites [OCM; with OCM (+OCM) or without (−OCM)] ^2^ from d 0 to 63 of gestation on the protein abundance of solute carrier family 2 member 3 (SLC2A3) in the fetal jejunum at 161 days of gestation.

			Supplementation				*p*-Values			
Area	Nut		−OCM	+OCM	SEM ^3^	Nut ^4^	SEM ^5^	Nut	OCM	Nut × OCM
Villus	CON		13.08	13.79	1.81	13.44	1.28	0.08	0.42	0.23
	RES		18.36	14.78		16.57				
		OCM ^6^	15.72	14.28						
Crypt	CON		1.69	1.04	0.15	1.37	0.10	0.97	<0.0001	0.84
	RES		1.72	1.02		1.36				
		OCM	1.71	1.03						
Total	CON		14.77	14.84	1.84	14.81	1.30	0.08	0.24	0.23
	RES		20.09	15.80		17.95				
		OCM	17.43	15.32						

Data are presented as relative fluorescence units. ^1^ CON (0.45 kg·heifer^−1^·d^−1^); RES = (−0.23 kg·heifer^−1^·d^−1^). ^2^ +OCM = (with one-carbon metabolites supplementation); –OCM = (without one-carbon metabolites supplementation). ^3^ Average SEM for nutritional levels × OCM supplementation interaction [CON − OCM (*n* = 7); CON + OCM (*n* = 8); RES − OCM (*n* = 7); RES + OCM (*n* = 7)]. ^4^ Main effect of nutritional levels. ^5^ Average SEM for Nut and OCM. ^6^ Main effect of OCM supplementation.

**Table 6:** The effect of maternal nutritional levels [Nut; control (CON) or restricted (RES)] ^1^ and supplementation with one-carbon metabolites [OCM; with OCM (+OCM) or without (−OCM)] ^2^ from d 0 to 63 of gestation on the protein abundance of solute carrier family 2 member 5 (SLC2A5) in the fetal jejunum at 161 days of gestation.

			Supplementation				*p*-Values			
Area	Nut		−OCM	+OCM	SEM ^3^	Nut ^4^	SEM ^5^	Nut	OCM	Nut × OCM
Villus	CON		2.55 ^a^	2.56 ^a^	0.36	2.56	0.25	0.19	0.04	0.05
	RES		2.30 ^a^	3.79 ^b^		3.04				
		OCM ^6^	2.42	3.18						
Crypt	CON		2.46	2.29	0.23	2.38	0.15	0.22	0.03	0.17
	RES		2.49	1.71		2.10				
		OCM	2.48	2.00						
Total	CON		4.94	4.86	0.39	4.90	0.25	0.46	0.39	0.28
	RES		4.80	5.51		5.15				
		OCM	4.87	5.18						

Data are presented as relative fluorescence units. ^1^ CON (0.45 kg·heifer^−1^·d^−1^); RES = (−0.23 kg·heifer^−1^·d^−1^). ^2^ +OCM = (with one-carbon metabolites supplementation); –OCM = (without one-carbon metabolites supplementation). ^3^ Average SEM for nutritional levels × OCM supplementation interaction [CON − OCM (*n* = 7); CON + OCM (*n* = 8); RES − OCM (*n* = 7); RES + OCM (*n* = 7)]. ^4^ Main effect of nutritional levels. ^5^ Average SEM for Nut and OCM. ^6^ Main effect of OCM supplementation. Lowercase letters indicate significant differences (*p* ≤ 0.05).

**Table 7:** The effect of maternal nutritional levels [Nut; control (CON) or restricted (RES)] ^1^ and supplementation with one-carbon metabolites [OCM; with OCM (+OCM) or without (−OCM)] ^2^ from d 0 to 63 of gestation on the protein abundance of solute carrier family 5 member 1 (SLC5A1) in the fetal jejunum at 161 days of gestation.

			Supplementation				*p*-Values			
Area	Nut		−OCM	+OCM	SEM ^3^	Nut ^4^	SEM ^5^	Nut	OCM	Nut × OCM
Villus	CON		4.44 ^b^	8.24 ^a^	0.87	6.34	0.61	<0.0001	0.05	0.01
	RES		10.21 ^a^	9.80 ^a^		10.00				
		OCM ^6^	7.32	9.02						
Crypt	CON		4.33 ^a^	4.66 ^a^	0.34	4.50	0.24	0.005	0.20	0.02
	RES		4.14 ^a^	2.96 ^b^		3.55				
		OCM	4.24	3.81						
Total	CON		8.77 ^b^	12.91 ^a^	0.95	10.84	0.67	0.004	0.17	0.003
	RES		14.35 ^a^	12.76 ^a^		13.56				
		OCM	11.56	12.83						

Data are presented as relative fluorescence units. ^1^ CON (0.45 kg·heifer^−1^·d^−1^); RES = (−0.23 kg·heifer^−1^·d^−1^). ^2^ +OCM = (with one-carbon metabolites supplementation); –OCM = (without one-carbon metabolites supplementation). ^3^ Average SEM for nutritional levels × OCM supplementation interaction [CON − OCM (*n* = 7); CON + OCM (*n* = 8); RES − OCM (*n* = 7); RES + OCM (*n* = 7)]. ^4^ Main effect of nutritional levels. ^5^ Average SEM for Nut and OCM. ^6^ Main effect of OCM supplementation. Lowercase letters indicate significant differences (*p* ≤ 0.05).

## Data Availability

The raw data supporting the conclusions of this article will be made available by the authors on request.
